# pH-Responsive supramolecular vesicles for imaging-guided drug delivery: Harnessing aggregation-induced emission

**DOI:** 10.1098/rsos.240664

**Published:** 2024-09-25

**Authors:** Xin-Rui Wang, Wei-Xiu Lin, Yi-Long Lu, Dietmar Kuck, Wen-Rong Xu

**Affiliations:** ^1^ School of Chemistry and Chemical Engineering, Key Laboratory of Advanced Materials of Tropical Island Resources of Ministry of Education, Hainan Provincial Key Laboratory of Fine Chemistry, Hainan University, Haikou 570228, People’s Republic of China; ^2^ Department of Chemistry and Center for Molecular Materials (CM2), Bielefeld University, Bielefeld 33615, Germany

**Keywords:** supramolecular vesicles, pH-responsive, drug delivery, aggregation-induced emission, imaging-guided

## Abstract

The water-soluble tribenzotriquinacene-based hexacarboxylic acid ammonium salt, **TBTQ-C**_**6**_, acts as the host component (**H**) forming host–guest complexes with tetraphenylethylene (TPE)-functionalized monotopic and tetratopic quaternary ammonium derivatives, **G1** and **G2**, to yield supra-amphiphiles. These supra-amphiphiles self-assemble to form pH-responsive fluorescent vesicles, which have allowed us to capitalize on the aggregation-induced emission (AIE) effect for imaging-guided drug delivery systems. These systems exhibit efficient drug loading and pH-responsive delivery capabilities. Upon encapsulation of the anticancer drug doxorubicin (DOX), both the TPE and DOX chromophores undergo dual-fluorescence deactivation due to the energy transfer relay (ETR) effect. Under acidic conditions, the release of DOX interrupts the ETR effect, resulting in the fluorescence recovery of TPE fluorogens and DOX, allowing for real-time visual monitoring of the drug release process. Cytotoxicity experiments confirmed the low toxicity of the unloaded vectors to normal cells, while the DOX-loaded vectors were found to significantly enhance the anticancer activity of DOX against cancer cells *in vitro*. The AIE-featured supramolecular vesicles presented in this research hold great potential for imaging-guided drug delivery systems.

## 1. Introduction

The self-assembly of amphiphilic molecules in aqueous environments has garnered increasing attention due to its significance in elucidating and emulating key processes within natural systems [1–4]. Unlike conventional amphiphiles, supra-amphiphiles [5–8], which align their hydrophilic and hydrophobic segments through noncovalent interactions or dynamic covalent bonds, offer a substantial reduction in the effort required for chemical synthesis. Moreover, supra-amphiphiles serve as building blocks for creating a diverse array of highly ordered nano-architectures, such as vesicles, micelles, tubes and ribbons [9–12]. Owing to the dynamics of reversible aggregation, the architectures of supra-amphiphiles can be controlled in various ways by external stimulation, such as variations in pH, exposure to light, temperature fluctuations, redox conditions and enzymatic reactions [13–15].

**Scheme 1.**
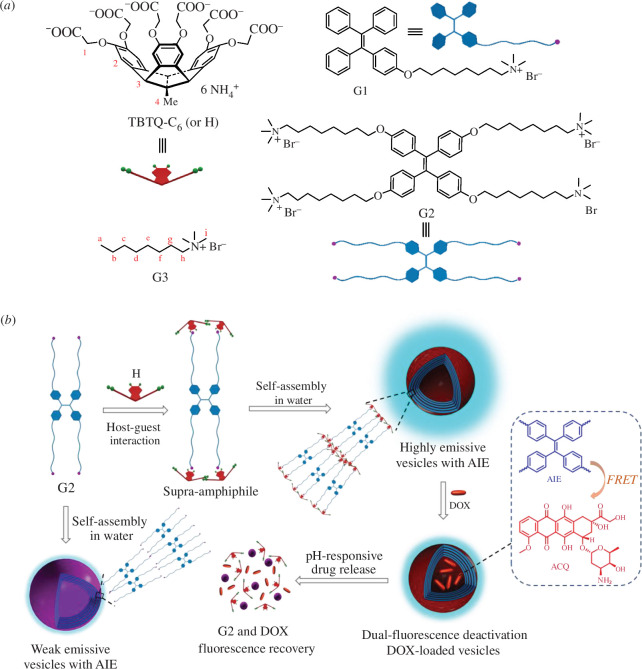
(*a*) Chemical structures and symbols of the host compound **TBTQ-C_6_** (or **H**) and guest components **G1**, **G2** and **G3**. (*b*) Schematic illustration of the formation of the fluorescent supramolecular vesicles **H-G2** and the pH-responsive drug upload and release.

Over the past few decades, macrocyclic receptors such as crown ethers, cyclodextrins, calixarenes, cucurbiturils, pillararenes and cyclotriveratrylenes have been extensively designed and synthesized to investigate their interactions with guest molecules. This research facilitates the formation of supra-amphiphiles and assesses their suitability for molecular encapsulation studies [16–18]. Tribenzotriquinacene (TBTQ) and its multifaceted derivatives, notable for their rigid, tripodal bowl-shaped skeleton composed of three mutually orthogonal indane wings, are considered effective host molecules. Similar to the aforementioned macrocycles, they are adept at binding guest molecules. However, unlike those macrocycles, the closed molecular bottom of TBTQ derivatives can effectively prevent guest threading, thereby establishing a more secure and enclosed environment for guest molecules. This not only minimizes the possibility of guest leakage or escape but also reduces unwanted interactions between the encapsulated guest and the exterior environment. Moreover, the facile incorporation of functional groups onto the peripheral aromatic rings of the TBTQ framework facilitates the expansion of its initially small and shallow cavity, thereby augmenting its diversity and functional potential [19]. Numerous host–guest studies focusing on TBTQ derivatives, conducted in both organic [20–25] and aqueous media [26–30], have been carried out by our research group and other scientific teams. In our recent publications, we successfully synthesized two water-soluble hexacarboxylated tribenzotriquinacene derivatives, **TBTQ-C**_**6**_ [27] (or **H**, scheme 1) and **TBTQ-CB6** [28]. The former has shown potential in forming pH- and photo-responsive supramolecular architectures [27,29], and in offering protection against acetylcholine hydrolysis [30], while the latter has proven to be effective as a drug carrier at the molecular level [28]. Motivated by the biomedical potential of these derivatives, further investigations have been initiated to create ‘smart’ supramolecular vesicles derived from stimuli-responsive supra-amphiphiles, due to their promising applications in drug delivery systems (DDS) [31–34]. However, current DDS often lack visibility and are difficult to track once inside cells and after drug release. Therefore, it is crucial to develop ‘visible’ nanocarriers that enable the monitoring of drug distribution both *in vitro* and *in vivo* [35–38].

To address this challenge, we have turned to a new class of organic luminogens exhibiting aggregation-induced emission (AIE) characteristics [39–41], which, contrary to the common aggregation-caused quenching (ACQ) effect observed in traditional fluorophores [35,42], enhance fluorescence upon aggregation. Tetra-phenylethylene (TPE) stands out among these luminogens for its simple structure and remarkable AIE properties, making it an exceptional candidate for biological labelling and imaging [43–45]. Building on these insights, our most recent work has pioneered the development of pH-responsive fluorescent supramolecular nanoparticles by utilizing **TBTQ-C_6_** and TPE-labelled chitosan-based supra-amphiphiles. These nanoparticles are engineered to maintain stability in acidic conditions and to disassemble in alkaline environments, thereby offering significant potential for use in visually trackable oral DDS [29].

In the work presented here, **TBTQ-C_6_** served as the host, and TPE-functionalized monotopic and tetratopic quaternary ammonium derivatives, **G1** and **G2**, were employed as guests and luminogens to generate **H-G1** (refer to electronic supplementary material, scheme S1) and **H-G2** supra-amphiphiles via host–guest interactions in water (scheme 1). These supra-amphiphiles were further converted into pH-responsive fluorescent supramolecular vesicles, which were then used as DDS for encapsulating doxorubicin (DOX), a widely utilized chemotherapeutic agent and model anticancer drug [46]. This approach established a dual-fluorescence quenched Förster resonance energy transfer (FRET) system, with the TPE entities acting as donor fluorophores and the DOX units as acceptors. The supramolecular vesicles showed robust stability in aqueous solutions of pH levels above 7.0. However, upon decrease of the pH below 7.0, the host–guest recognition ceased, leading to the disassembly of the supra-amphiphiles and subsequent drug release along with vesicle collapse. Consequently, this interruption of the energy transfer relay (ETR) effect between TPE and DOX led to the recovery of fluorescence, enabling real-time visual monitoring of the drug loading and release processes. Cellular uptake and cytotoxicity experiments confirmed the low toxicity of the vesicle carriers and their ability to maintain the inhibitory effect of DOX on cancer cells. Therefore, such TBTQ-based supramolecular DDS with prominent pH-responsiveness and indicative emission changes may hold promise for applications in cancer treatment.

## 2. Experimental section

### 2.1. Materials and methods

Doxorubicin hydrochloride (DOX, 98%) was purchased from Aladdin Reagent Co., Ltd. All commercially available reagents were used as received unless specified otherwise. The ^1^H NMR and ^13^C NMR spectra were recorded on a 400 MHz Bruker NMR spectrometer and chemical shifts were reported in ppm. Mass spectra were recorded using electrospray ionization (ESI) with an LCMS-IT-TOF instrument (Shimadzu, Japan). The determination of the critical aggregation concentration (CAC) values was carried out on a DDS-307 precision digital conductivity meter (Shanghai Leici, China). Transmission electron microscopy (TEM) investigations were carried out on a JEM-1200EX microscope (Jeol Ltd., Japan). Dynamic light scattering (DLS) and ζ-potential were measured on a Zetasizer Nano S90 particle size analyzer (Malvern Instruments, England) at room temperature. UV-vis spectra were taken on a UV-1800 UV-vis spectrophotometer (Shimadzu, Japan), and fluorescence spectra were recorded on a F97pro fluorescence spectrophotometer (Lengguang, China). Fluorescence in cells was studied and tracked by a confocal laser scanning microscope (CLSM, Leica TCS SP8, Germany).

### 2.2. Synthetic procedures

Synthesis of **TBTQ-C**_**6**_, **G1** and **G2**, **TBTQ-C**_**6**_ and **G1** were synthesized in the preceding work [27,47]. The synthesis and characterization of compound **G2** are given in the electronic supplementary material.

### 2.3. DOX loading of H-G1 vesicles and H-G2 vesicles

A certain amount of DOX was added to a freshly prepared aqueous solution of **H** and **G** (1.0 mM for both) to prepare DOX-loaded vesicles. The final concentrations of DOX, **H** and **G** were 0.15, 1.0 and 1.0 mM, respectively. Then the resulting DOX-loaded vesicles were purified by dialysis for several times through a dialysis bag with a molecular weight cut-off of 1000 in distilled water, until the water outside the dialysis bag showed negligible DOX absorption. Consequently, DOX-loaded **H-G1** and **H-G2** vesicles were successfully prepared. The DOX encapsulation efficiencies were calculated by the following equation [48]:


EncapsulationEfficiency(%)=(mDOX−loaded/mDOX)×100

where mDOX-loaded and mDOX are the mass of DOX encapsulated in vesicles and the mass of DOX added, respectively. To calculate mDOX-loaded, the pH of the vesicle solutions loaded with DOX was adjusted to 1.0 to ensure the complete release of DOX. The mass of DOX was determined by UV spectrophotometry at 480 nm and calculated relative to a standard calibration curve at a concentration of 0.025–0.25 mM in water [49].

### 2.4. Controllable DOX release *in vitro*

Buffer solutions with pH = 7.4, 5.5 and 4.0 were prepared with citric acid and sodium dihydrogen phosphate as drug release medium to simulate normal physiological conditions and tumour cell conditions. In a general release experiment, the volume of 7.00 ml of DOX-loaded vesicles was adjusted to 10.00 ml with an appropriate release medium at 37°C. In the selected time intervals, an aliquot of 3 ml of the release solution was taken out to measure the released DOX concentrations by UV–vis method, and then it was poured back into the original release solution. The concentration of DOX was determined by measuring the absorbance at 480 nm using a standard absorbance–concentration curve of DOX in the corresponding release buffer.

### 2.5. Cell culture

L02 normal cells (human liver cell line) and HepG2 cells (a human hepatoma cell line) were selected for cell experiments. The cells were cultivated in Dulbecco’s modified Eagle’s medium (DMEM) supplemented with 10% fetal bovine serum and 1% antibiotics (10 kU ml^−1^ penicillin and 10 mg ml^−1^ streptomycin in a humid environment with 5% CO_2_ at 37°C).

### 2.6. Cytotoxicity assay

The CCK-8 assay was used to evaluate the relative cytotoxicity of **H**, **G1** and **G2**, as well as the **H-G1** and **H-G2** vectors against L02 normal cells, and DOX, DOX-loaded **H-G1** vectors and DOX-loaded **H-G2** vectors against HepG2 cells *in vitro*. Initially, cells were seeded at a density of 10 000 cells per well in 96-well plates containing 200 μl DMEM, and cultured under 5% CO_2_ at 37°C for 24 h. Subsequently, testing samples of **H**, **G1**, **G2**, **H-G1** vectors, **H-G2** vectors, DOX, DOX-loaded **H-G1** and DOX-loaded **H-G2** vectors, were first dissolved in the culture medium to give stock solutions which were further diluted with the culture medium for cytotoxicity studies. The L02 normal cells or HepG2 cells were incubated with 200 μl of the above-diluted solutions under 5% CO_2_ at 37°C for 72 h. Then the medium containing the drug solutions was removed and the cells were incubated for another 1 h by adding 100 μl CCK-8. Wells containing the same amount of cell culture medium and CCK-8 solution but lacking cells and testing drugs served as blank groups, while wells containing cells, the same amount of cell culture medium and CCK-8 solution without adding drugs were designated as control groups. After shaking the plates for 1 min, the absorbance of the formazan product was measured using a microplate reader (BioTek ELx808) at 450 nm. The mean optical density (OD, absorbance) of three wells in the indicated groups was used to calculate the percentage of cell viability using the following equation:


$$Cell\;Viability\;(\%)=\left(A_{sample}-A_{blank}\right)/\left(A_{control}-A_{blank}\right)\times 100$$

where *A*_sample_, *A*_blank_ and *A*_control_ denote the measured absorbance of the samples, blank groups and control groups, respectively.

### 2.7. Cellular uptake and intracellular localization

The cellular uptake and intracellular localization of **H-G1** vectors, **H-G2** vectors, DOX and the DOX-loaded vectors were examined in HepG2 cancer cells. In brief, the cells were seeded in Ø35 mm glass bottom dishes for 24 h prior to treatment to allow them to adhere and reach an optimal density for subsequent treatments. This ensures uniformity and reproducibility in the cellular responses. Then the cells were treated with **H-G1** vectors, **H-G2** vectors and DOX at 37°C for 8 h, and with DOX-loaded **H-G1** and DOX-loaded **H-G2** vectors at 37°C for 1, 4, 8 and 12 h. After rinsing with phosphate-buffered saline (PBS), the cell nuclei were stained with 400 μl of a solution containing SiR-Hoechst (10 μΜ) and verapamil (10 μΜ) for 1 h, followed by another rinse with 2 ml of PBS. The fluorescence characteristics of the samples were investigated by CLSM. To examine the cellular uptake of samples, the excitation filter was set to 373 nm and the emission filter to 484 nm for **H-G1** vectors; for **H-G2** vectors, the excitation filter was set to 384 nm and the emission filter to 486 nm; for SiR-Hoechst, the excitation filter was 633 nm and the emission filter was 674 nm; for DOX, the excitation filter was 469 nm and the emission filter was 596 nm.

## 3. Results and discussion

### 3.1. Host–guest complexation studies in water

**Figure 1.**
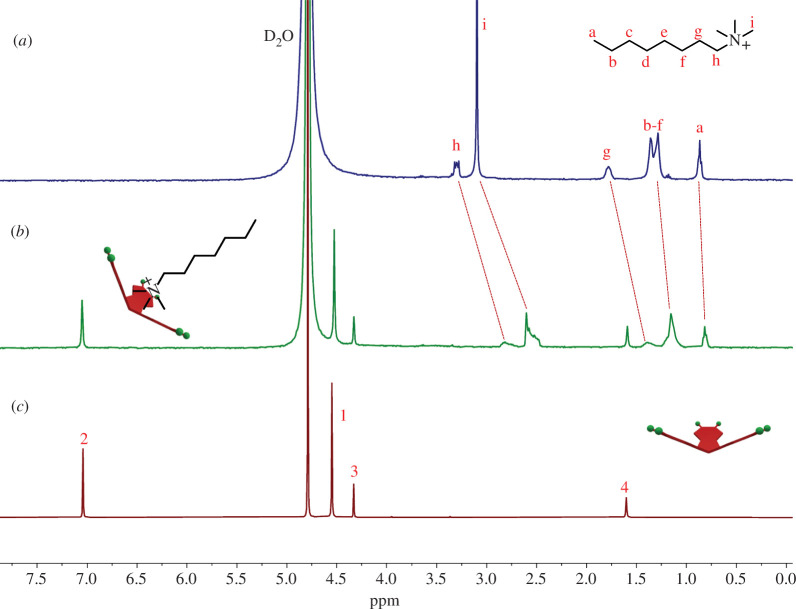
^1^H NMR spectra (400 MHz, D_2_O, 25°C) of (*a*) **G3** alone; (*b*) **G3** and **H**; (*c*) **H** alone ([**G3**] = [**H**] = 3.00 mM).

The host compound **TBTQ-C_6_** [27] and guest compound **G1** [47] were synthesized following previously reported methods. Guest compound **G2** was prepared from tetrakis-(4-hydroxyphenyl)-ethene [50] through a fourfold Williamson ether synthesis followed by the creation of four terminal ammonium ion centres (scheme S2). The new compounds were comprehensively characterized by NMR spectroscopy and mass spectrometry (see electronic supplementary material). Before investigating the self-assembly processes of these compounds, ^1^H NMR spectroscopy was used to examine the host–guest association characteristics between **TBTQ-C_6_** and the ammonium guests. Due to the insufficient water solubility of **G1** and **G2** on their part, a model compound, *N*,*N*,*N*-trimethyl-*n*-octylammonium bromide (**G3**, scheme 1), was used for these experiments. As illustrated in figure 1, the proton signals associated with H^a^, H^b–f^, H^g^, H^h^ and H^i^ of guest **G3** experienced significant upfield shifts (Δδ = –0.06, –0.17, –0.40, –0.49 and –0.52 ppm, respectively) and broadening in the presence of the equimolar amounts of **TBTQ-C_6_**. This shift is attributed to the shielding effect of the electron-rich internal cavity within the tribenzotriquinacene framework [51], signifying the close coordination of the cationic component of **G3** to the concave molecular surface of the host **TBTQ-C_6_**. The broadening of the signals may result from restricted rotation within the complex or from the dynamics of the complexation. At the same time, the proton resonances of H^1^, H^2^ and H^4^ of **TBTQ-C_6_** exhibited only very slight shifts (Δδ = –0.02, +0.01 and –0.01 ppm, respectively) but also became significantly broadened, which also provides evidence for the successful complexation between **TBTQ-C_6_** and **G3**.

Isothermal titration calorimetry (ITC) experiments were conducted to ascertain the association constant (*K*_a_) and stoichiometry of the host–guest complexes. The *K*_a_ value was determined to be (3.28 ± 1.11) × 10^5^ M^–1^ for **H-G1** in a 1 : 1 binding ratio (*n* = 1.063), as presented in electronic supplementary material, figure S6, and (4.27 ± 0.86) × 10^3^ M^–1^ for **H-G2** in a 4 : 1 binding ratio (*n* = 0.231), as shown in electronic supplementary material, figure S7. These findings validate the successful formation of the inclusion complexes **H-G1** and **H-G2** in aqueous solutions. It is reasonable to infer that these complexes give rise to supra-amphiphiles, wherein the **TBTQ-C_6_** residue exhibits hydrophilicity and the alkyl-TPE residues of **G1** and **G2** demonstrate hydrophobicity, as depicted in scheme 1 and electronic supplementary material, S1.

The pH-responsive nature of TBTQ-based hexacarboxylate **TBTQ-C_6_**, as documented previously [27], motivated us to explore the pH-responsive behaviour of the complexes **H-G1** and **H-G2** using ^1^H NMR spectroscopy. Before conducting these experiments, an examination of the model guest compound **G3** was performed. Upon the addition of DCl to acidify the **H-G3** solution, the water-soluble host **H** precipitated from D_2_O, resulting in the disappearance of its proton signals. Concurrently, the proton signals of **G3** returned to their positions prior to association with **H**. This unequivocally indicated the disruption of the host–guest complex (electronic supplementary material, figure S8). Conversely, when NaOD was added to alkalize the solution, the precipitated host redissolved and its proton signals reappeared. Simultaneously, the proton resonances of **G3** shifted upfield again. These observations undeniably confirm the reversible pH-responsive complexation between the host **TBTQ-C_6_** and the guest **G3**.

### 3.2. Construction of fluorescent supramolecular vesicles

**Figure 2.**
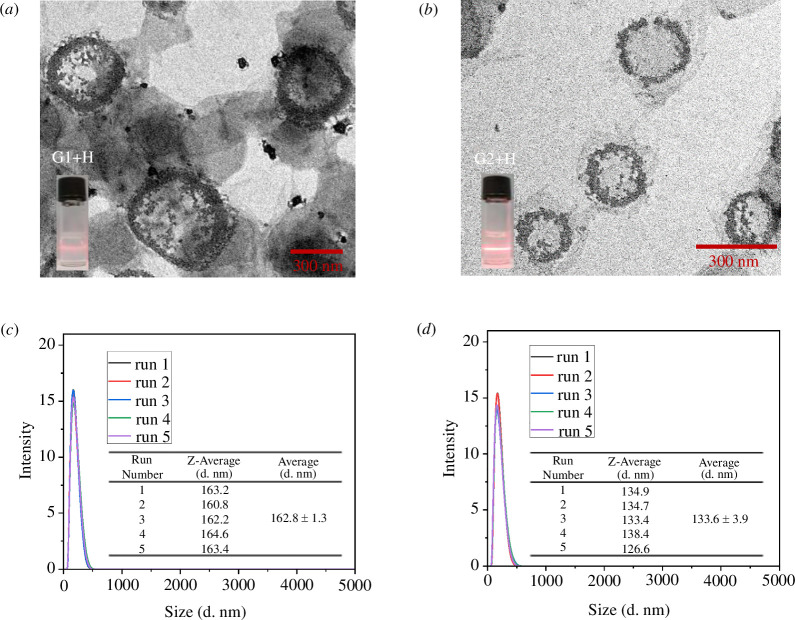
TEM images of (*a*) **H-G1** and (*b*) **H-G2** aggregates (scale bars = 300 nm, insets show the corresponding Tyndall effect). DLS particle size distribution of (*c*) **H-G1** and (*d*) **H-G2** aggregates for five repeat runs (insets show *Z*-average size and average ± s.d.). ([**H**]/[**G1**] = 1 : 1, [**H**]/[**G2**] = 4 : 1, [**G1**] = [**G2**] = 7.82 × 10^–4^ M, in water).

After establishing the host–guest recognition of the **H-G1** and **H-G2** motifs, the subsequent self-assembly behaviour of these complexes in water was investigated. Primarily, the critical aggregation concentrations (CACs) of **G1**, **H-G1**, **G2** and **H-G2** were determined by concentration-dependent conductivity measurements, yielding values of 6.33 × 10^–4^, 7.53 × 10^–4^, 5.81 × 10^–4^ and 4.35 × 10^–4^ M, respectively (electronic supplementary material, figure S9). Notably, the individual solutions exhibited distinct Tyndall effects above the CACs, suggesting the formation of substantial nanoparticles (figure 2 and electronic supplementary material, figure S10). Transmission electron microscopy (TEM) and dynamic light scattering (DLS) were used to examine the morphology and size distribution of these aggregates. The solution of guest **G1** formed micelles with an average diameter of 711 nm, as evident in the TEM image (electronic supplementary material, figure S10). Intriguingly, upon the addition of 1.00 equivalent of host **TBTQ-C_6_**, uniform vesicles with an average diameter of 315 nm and a wall thickness of 50 nm were observed through TEM measurements (figure 2*a*). We hypothesize that the **H-G1** supra-amphiphiles first self-assemble to form a bilayer structure, which then bends to generate multilamellar spheres, as illustrated in electronic supplementary material, scheme S1. Notably, the fourfold-tailed analogue **G2**, when alone, was observed to create vesicles with a diameter of 431 nm and a wall thickness of 62 nm (electronic supplementary material, figure S10), a morphology distinct from that of the single-tail compound **G1** in isolation. The varying morphologies of **G1** and **G2** may provide insights into the potential for designing the transition from micelles to vesicles by increasing the hydrophilic groups of the guest constituent [52]. Upon the addition of the host **TBTQ-C_6_**, uniform **H-G2** vesicles with an average diameter of 282 nm were observed (figure 2*b*). The wall thickness of the **H-G2** vesicles was found to be 20 nm, indicating the formation of a multilayer structure (scheme 1b) similar to that of the **H-G1** vesicles. The sizes of the aggregates obtained by DLS measurements were found to be 465.5 ± 2.0 nm (electronic supplementary material, figure S10b), 194.8 ± 1.7 nm (electronic supplementary material, figure S10d), 162.8 ± 1.3 nm (figure 2*c*) and 133.6 ± 3.9 nm (figure 2*d*) for **G1**, **G2**, **H-G1** and **H-G2**, respectively, across five repeat measurements. Interestingly, the DLS diameters are smaller than the TEM results, which might be because DLS measures the hydrodynamic diameter of particles in their solvated state, including the solvation shell. In contrast, TEM measures the dry particle size, which can appear larger due to potential aggregation during the drying process. Additionally, DLS can be influenced by the presence of smaller particles or fragments that contribute to a lower average size, whereas TEM provides a direct visual representation of individual particles. Moreover, the ζ-potential measurements provide insight into the surface charge and stability of the **H-G1** and **H-G2** aggregates (electronic supplementary material, figure S11). For **H-G1** aggregates (electronic supplementary material, figure S11a), the average ζ-potential across five runs was –29.6 ± 0.3 mV, indicating a stable negative surface charge that helps maintain colloidal stability by preventing aggregation. Similarly, **H-G2** aggregates (electronic supplementary material, figure S11b) exhibited an average ζ-potential of –35.0 ± 1.0 mV, suggesting slightly higher colloidal stability compared to **H-G1** due to stronger electrostatic repulsion [53–55]. The consistent negative zeta potential values for both aggregates are crucial for maintaining dispersion in aqueous solutions, which is essential for applications such as drug delivery. The fluorescence characteristics of these supramolecular vesicles were also examined. As shown in electronic supplementary material, figure S12, the individual emissions of **G1** and **G2** displayed relatively low intensity, but a notable increase in emission was observed upon the introduction of **H**. This enhancement can be attributed to the restriction of the intramolecular rotation of the phenyl rings in **G1** and **G2**, brought about by the formation of the **H-G1** and **H-G2** supra-amphiphile-based vesicles [56].

### 3.3. DOX encapsulation and *in vitro* release

DOX served as the model drug for investigating the encapsulation efficiency and release performance of the **H-G1** and **H-G2** vesicles. To create the DOX-loaded vesicles, aqueous solutions of DOX were rapidly introduced to freshly prepared solutions of **H** and **G1** ([**H**]/[**G1**] = 1 : 1) or of **H** and **G2** ([**H**]/[**G2**] = 4 : 1). The resulting mixtures underwent purification through dialysis to yield DOX-loaded **H-G1** vesicles and DOX-loaded **H-G2** vesicles, respectively. According to the UV absorbance of DOX at 480 nm, the DOX loading efficiency was calculated to be 14.7% for **H-G1** vesicles and 19.8% for **H-G2** vesicles, suggesting a good drug-loading capability well above the average level of supramolecular vesicles [57]. Supramolecular vesicles demonstrate effective loading of DOX, primarily attributed to their amphiphilic nature resulting from the multilayer self-assembly of supra-amphiphiles in aqueous environments. Within this structure, hydrophilic ends face the aqueous phase while hydrophobic ends are positioned away from it. This configuration endows supramolecular vesicles with both a hydrophilic outer layer and a hydrophobic inner layer, facilitating encapsulation of both water-soluble and hydrophobic drugs. Furthermore, the internal cavity structure of supramolecular vesicles allows for accommodation of drug molecules. Additionally, the surface charge density of supramolecular vesicles also affects their drug-loading efficiency. The enhanced loading efficiency observed in **H-G2** vesicles is likely due to their slightly more negative surface charge density and greater stability, as shown by the ζ-potential results. Because of the positively charged nature of DOX hydrochloride, it can be more effectively encapsulated within the negatively charged supramolecular vesicles through electrostatic interactions.

**Figure 3.**
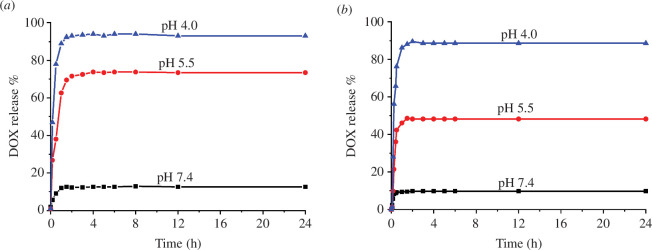
pH-Responsive DOX release profiles of (*a*) the DOX-loaded **H-G1** vesicles and (*b*) the DOX-loaded **H-G2** vesicles in aqueous solution of different pH values.

The controlled drug release behaviour of DOX-loaded vesicles was studied *in vitro* by adjusting the pH of the solution. As shown in figure 3, the cumulative release of DOX in nearly neutral conditions (pH 7.4) was only about 12.5% for **H-G1** vesicles and 9.7% for **H-G2** vesicles within 24 h. This indicates that the vesicles exhibit exceptional stability, effectively preventing leakage under physiological conditions. However, the vesicles demonstrated efficient and rapid release of DOX in acidic environments with pH levels of 5.5 and 4.0, achieving 73.5% and 93.0% release for DOX-loaded **H-G1** vesicles, and 48.2% and 88.7% release for DOX-loaded **H-G2** vesicles, respectively. This enhanced release can be fundamentally linked to the protonation of carboxylate anions in the host molecule **H** when exposed to acidic media. Such protonation undermines the electrostatic interactions between the carboxylate anions of the host and the quaternary ammonium cations of the guests, leading to the breakdown of the supra-amphiphilic structure and subsequent vesicle disintegration. As a result, a decrease in pH correlates with a marked increase in the release rate of DOX.

**Figure 4.**
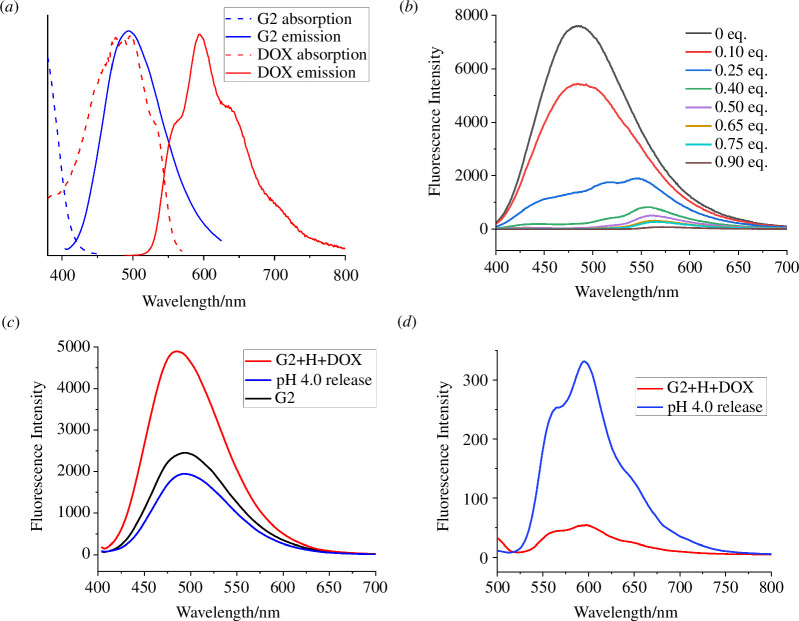
(*a*) Absorption and fluorescence emission spectra of **G2** and DOX. (*b*) Fluorescence spectra of **H-G2** vesicles in the presence of different amounts of DOX (*λ*_ex_ = 384 nm). (*c*) Fluorescence recovery of **G2** after adjustment of the pH of DOX-loaded **H-G2** vesicles (**G2**+**H**+**DOX**) to 4.0 (*λ*_ex_ = 384 nm); (*d*) fluorescence recovery of DOX after adjustment of the pH of DOX-loaded **H-G2** vesicles (**G2**+**H**+**DOX**) to 4.0 (*λ*_ex_ = 469 nm) ([**H**]/[**G2**] = 4 : 1, [**G2**] = 1.0 mM).

Imaging-guided DDS systems, which involve dynamic energy transfer mechanisms like Förster resonance energy transfer (FRET) [58–61] between the drug and the carrier, have attracted substantial interest. Upon the drug’s release in targeted cellular environments, these energy transfers are interrupted, resulting in alterations to the fluorescent signal. This signal change serves to monitor the translocation, drug release and elimination of the nanomedicine. Notably, the emission spectra of **G1** and **G2** significantly overlap with the absorption spectrum of DOX (figure 4*a*), suggesting that **G1** and **G2** could serve as fluorescent donors for DOX. To confirm the FRET phenomenon, **G2** was selected for a test. The fluorescence of **H-G2** vesicles is gradually quenched when increasing the amount of DOX (figure 4*b*), suggesting that the AIE behaviour of **G2** disappeared due to the FRET from TPE-based fluorogens to DOX. In addition, the fluorescence of the DOX molecules was self-quenched due to the ACQ effect through π–π stacking of rigid planar aromatic rings. Thus, the energy transfer relay (ETR) effect gives rise to dual-fluorescence-quenched supramolecular systems [35]. As the pH of the solution was lowered to 4.0, the host–guest interactions weakened, which led to the decomposition of the supramolecular vesicles and to the release of DOX. Therefore, the ETR effect operating between **G2** and DOX ceased upon release of the drug, leading to the fluorescence recovery of **G2** (figure 4*c*) and DOX (figure 4*d*).

### 3.4. Cellular uptake and *in vitro* cytotoxicity

**Figure 5.**
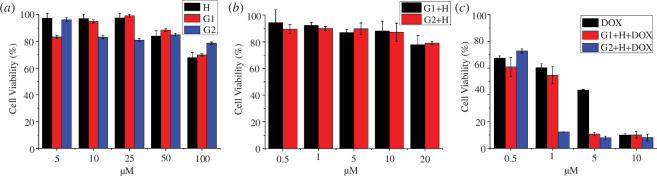
Cytotoxicity of different concentrations of (*a*) **H**, **G1** and **G2**; (*b*) **H-G1** ([**H**]/[**G1**] = 1 : 1, [**G1**] = 0.5, 1, 5, 10 and 20 μM) and **H-G2** ([**H**]/[**G2**] = 4 : 1, [**G2**] = 0.5, 1, 5, 10 and 20 μM) vectors against L02 normal cells after incubated for 72 h; (*c*) the cytotoxicity of different concentrations of DOX, DOX-loaded **H-G1** and **H-G2** vectors (with [DOX] = 0.5, 1, 5 and 10 μM) against HepG2 cells after incubated for 72 h.

Biocompatibility is a significant parameter for assessing the suitability of a drug delivery system in biomedical applications [62]. Consequently, a basic evaluation of the cytotoxicity of **H**, **G1** and **G2**, as well as their complexes **H-G1** and **H-G2**, was conducted against L02 normal cells using the Cell Counting Kit-8 (CCK-8) assay [63]. Their toxicity was found to remain at a low level after incubation for 72 h, implying good biocompatibilities of these supramolecular nanocarriers (figure 5*a,b*). To further evaluate the anticancer performance of these drug delivery systems, HepG2 cancer cells were incubated with DOX and the DOX-loaded **H-G1** and **H-G2** complexes for 72 h. At concentrations exceeding 1.0 μM, the inhibitory effect of DOX on cell activity increased after loading the drug into the vectors, suggesting that encapsulating DOX with these drug delivery systems enhances the therapeutic effect of DOX (figure 5*c*).

**Figure 6.**
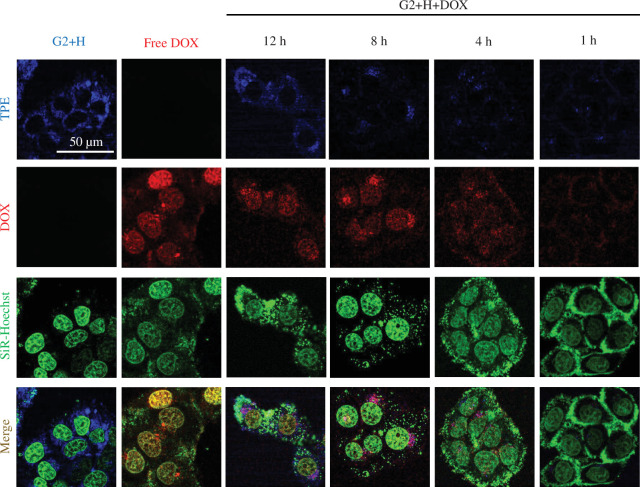
CLSM images of HepG2 cells treated with blank **G2+H**, free DOX and **G2**+**H**+**DOX** (DOX-loaded **H-G2** vectors). The cells were incubated with **G2**+**H** ([**H**]/[**G2**] = 4 : 1, [**G2**] = 20 μM) and DOX (5 μM) for 8 h, with DOX-loaded **H-G2** vectors ([DOX] = 5 μM) for 1, 4, 8 and 12 h.

To further investigate the cellular uptake and intracellular drug release behaviour of the DOX-loaded vectors in cancer cells, SiR-Hoechst was used to stain the DNA (mainly targeting the nuclei). The fluorescence patterns of DOX, **H-G1** vectors, DOX-loaded **H-G1** vectors (electronic supplementary material, figure S13), **H-G2** vectors and DOX-loaded **H-G2** vectors (figure 6) in HepG2 cells were then examined using confocal laser scanning microscopy (CLSM). Electronic supplementary material, figure S13 and figure 6 demonstrate that after incubation for 8 h, the fluorescence of **H-G1** and **H-G2** (blue) did not overlap with the green fluorescence of SiR-Hoechst and was concentrated around the green fluorescent areas. This indicated that **H-G1** and **H-G2** vectors were taken up by cells but did not enter the nucleus. Moreover, the blue fluorescence intensity of **H-G2** was higher than that of the **H-G1** system, suggesting its advantage for use as a visual drug carrier. As expected, the red fluorescence of free DOX was primarily located inside the nuclei, interacting with DNA to exert its cytotoxic effects. CLSM images of the DOX-loaded **H-G1** and **H-G2** vectors were captured at various incubation times. As depicted in figure 6, after 1 h of incubation, a faint blue fluorescence signal from **H-G2** and a red fluorescence signal from DOX were observed to overlap around the cell nuclei, indicating that the drug had not yet been released. With prolonged incubation, DOX gradually penetrated the cell nuclei, while the carrier remained localized around them. Both fluorescence signals notably intensified, suggesting that the rupture of supramolecular vesicles and drug release within cancer cells is time-dependent. This also confirms the interruption of the ETR effect upon drug release, allowing for the recovery of DOX and TPE fluorescence. In the **H-G1** drug delivery system, partial entry of DOX into the cell nuclei was already observed after 1 h of incubation (electronic supplementary material, figure S13), indicating a faster release of DOX by **H-G1** in cancer cells. This variation is likely attributable to the stability differences between the **H-G1** and **H-G2** carriers; the former’s lesser stability results in a quicker release of the drug in the acidic microenvironment of cancer cells, whereas the latter provides a more controlled, sustained release. These findings hint at the significant potential of the **H-G1** and **H-G2** systems, particularly the latter, in their roles as visual drug delivery vehicles for administering chemotherapeutic agents like DOX into cancer cells, offering therapeutic treatment and the capability for real-time monitoring of drug dissemination.

## 4. Conclusion

In conclusion, we have successfully developed novel host–guest supra-amphiphiles composed of a water-soluble tribenzotriquinacene hexacarboxylate (**TBTQ-C_6_**) as the host and two TPE-functionalized monotopic and tetratopic quaternary ammonium derivatives, **G1** and **G2**, as the guests. These supra-amphiphiles further self-assembled to generate highly emissive vesicles in water due to the AIE effect and were employed in an imaging-guided drug delivery system. The chemotherapy drug DOX was successfully encapsulated into the DDS with good loading efficiency, leading to significant dual-fluorescence-deactivation within the vesicles due to the ETR effect between TPE and DOX. The DDS also showed excellent drug release efficiency in an acidic environment *in vitro*. Notably, the ETR effect between TPE and DOX ceased upon DOX release, resulting in recovery in the fluorescence intensities of TPE fluorogens and DOX. The host **TBTQ-C_6_**, guest **G1** and **G2**, and supra-amphiphiles **H-G1** and **H-G2** all displayed low cytotoxicity. The encapsulation of DOX by their self-assembled vectors holds promise for enhancing the therapeutic potential of DOX in cancer cells. This tribenzotriquinacene-based supramolecular imaging-guided DDS shows significant potential in the field of cancer treatment.

## Data Availability

Data is available at Dryad [64]. Supplementary material is available online [65].
